# Effects on mobility training and de-adaptations in subjects with Spinal Cord Injury due to a Wearable Robot: a preliminary report

**DOI:** 10.1186/s12883-016-0536-0

**Published:** 2016-01-28

**Authors:** Patrizio Sale, Emanuele Francesco Russo, Michele Russo, Stefano Masiero, Francesco Piccione, Rocco Salvatore Calabrò, Serena Filoni

**Affiliations:** Department of Neurorehabilitation, I.R.C.C.S. San Camillo Hospital, via Alberoni 70, 30126 Venice, Italy; Fondazione Centri di Riabilitazione Padre Pio Onlus, San Giovanni Rotondo, Foggia, Italy; Department of Neuroscience, Rehabilitation Unit, University of Padua, Via Giustiniani 2, Padua, 35128 Italy; Neurobehavioral and Robotic Neurorehabilitation Laboratory Coordinator IRCCS Centro, Neurolesi “Bonino-Pulejo” Messina, Messina, Italy

**Keywords:** Spinal cord injury, Gait analysis, Rehabilitation, Robotics, Mobility, Lower limb

## Abstract

**Background:**

Spinal cord injury (SCI) is a severe neurological disorder associated not only with ongoing medical complications but also with a significant loss of mobility and participation. The introduction of robotic technologies to recover lower limb function has been greatly employed in the rehabilitative practice. The aim of this preliminary report were to evaluate the efficacy, the feasibility and the changes in the mobility and in the de-adaptations of a new rehabilitative protocol for EKSO™ a robotic exoskeleton device in subjects with SCI disease with an impairment of lower limbs assessed by gait analysis and clinical outcomes.

**Method:**

This is a pilot single case experimental A-B (pre-post) design study. Three cognitively intact voluntary participants with SCI and gait disorders were admitted. All subjects were submitted to a training program of robot walking sessions for 45 min daily over 20 sessions. The spatiotemporal parameters at the beginning (T0) and at the end of treatment (T1) were recorded. Other clinical assessments (6 min walking test and Timed Up and Go test) were acquired at T0 and T1.

**Results:**

Robot training were feasible and acceptable and all participants completed the training sessions. All subjects showed improvements in gait spatiotemporal indexes (Mean velocity, Cadence, Step length and Step width) and in 6 min Walking Test (T0 versus T1).

**Conclusions:**

Robot training is a feasible form of rehabilitation for people with SCI. Further investigation regarding long term effectiveness of robot training in time is necessary.

**Trial registration:**

ClinicalTrials.gov NCT02065830.

## Background

Spinal cord injury (SCI) is a severe neurological disorder associated not only with ongoing medical complications but also with a significant loss of participation [1]. The rehabilitation of SCI subjects focuses on recovering the highest possible level of autonomy and functioning. Mobility limitations are a key factor contributing to reduced function and reduced health and life satisfaction in the SCI population.

Mobility refers to any movements that lead to a change in position or location by one’s own means performed with or without technical assistance. Mobility allows us to carry out daily and domestic activities that are required in the various fields of human performance, such as personal care, work, education, leisure and play. The wheelchair can be the primary means of mobility for SCI subjects with a permanent or progressive disability [2].

Locomotor training has proved to provide a beneficial effect in terms of mobility in incomplete paraplegic patients. Improvement of locomotor activity occurs independently of the spontaneous recovery of the spinal cord function [3]. Several studies showed that spinal cord injury induced reorganization of the sensory and motor systems following rehabilitative training. During the era of modern rehabilitation, multiple compensatory techniques have been developed as potential substitutes for the residual neurological deficits that prevent a return to upright bipedal ambulation [4].

Wheeled vehicles, however, are excellent at reducing the effort of carrying substantial loads, but terrain and space restrictions often limit the practicality of a vehicle and require the versatility of legged locomotion [5]. Mechanical and electrical stimulation, robots and powered exoskeleton devices as well as combinations of these techniques have been used. In particular the locomotor training in SCI patients has been evolving over the last twelve years with the development of a new motorized robotic driven gait orthosis (DGO) and more recently, new exoskeletal systems allow patient mobilization outside the treadmill.

Over the last five years exoskeletal systems became available for SCI patients. Exoskeletons have also been developed to assist with unloaded locomotion. Exoskeletons for lower extremities have joints matching the patient’s lower limb joints and motors that drive movements over these joints to assist leg movements. These new wearable robots are mechanical suits known as exoskeletons that can help people with spinal cord injuries stand up and walk away from their wheelchairs. These robots were designed around the function and shape of the human body and the human is able to control the robotic limbs. This control could assist in walking, running, jumping higher or even lifting objects one would not normally be able to lift. Three exoskeletons (EKSO™, Rex® and ReWalk®) allow SCI patients to stand up, walk with a defined pattern and even climb stairs mainly on a basis of passive range of motion (ROM) [6–8]. The exoskeleton HAL® (Cyberdyne Inc., Japan) offers the possibility of being connected with the SCI patient through emg-electrodes on the skin at the extensor/flexor muscle region of the lower extremities. This allows voluntary machine supported ROM of incomplete SCI patients by using minimal bioelectrical signals recorded and amplified from hip and knee flexors and extensors [9, 10].

EKSO™ is a wearable bionic suit, which enables individuals with any amount of lower extremity weakness to stand up and walk over ground with a natural, full weight bearing, reciprocal gait. It is an exoskeleton that allows the wearer, regardless of the degree of difficulty in movement, to stand and move properly with all their body weight. Walking is achieved by the user’s weight shifts activating sensors in the device, which initiate steps. Battery-powered motors drive the legs, replacing deficient neuromuscular function. EKSO™ provides functional based rehabilitation, over ground gait training, and upright, weight bearing exercise unlike any other. It has been designed for the needs of busy therapists treating a wide range of patients in a single day. The suit is strapped over the users’ clothing with easy adjustments to transition between patients in as little as five minutes.

The aims of this preliminary report were to evaluate the efficacy, the feasibility and the changes in the mobility and in the de-adaptations of a new rehabilitative protocol for EKSO™, a robotic exoskeleton device in subjects with SCI disease with an impairment of lower limbs as assessed by gait analysis and clinical outcomes. The intent of this research is to develop a new evaluative and rehabilitative protocol and specifically, we wanted to explore the applicability of this system in patients with severely impaired gait function.

## Methods

This is a pilot single case experimental A-B (pre-post) design study. A preliminary medical examination included a physical and neurological test with a gait analysis. The following inclusion criteria were identified:

- chronic motor complete or incomplete cervical and thoracic (C7-T12) spinal cord injury;
- skin integrity;
- adequate hip, knee and ankle range of motion;
- spasticity level of 3 or less (Ashworth scale);
- ability to physically fit into the exoskeletal device;
- ability to tolerate upright standing for a minimum of 30 min;
- joint range of motion within normal functional limits for ambulation;
- sufficient upper body strength to balance themselves using the walker while wearing the exoskeleton.

The following exclusion criteria were identified:

i) Heart or respiratory comorbidity;
j) Hemodynamic instability;
k) Presence of unhealed fractures;
l) Presence of heterotopic ossification that may impede walking;
m) Presence of osteoporosis;
n) Height below 62 inches or above 74 inches;
o) Weight above 220 lbs;
p) Cognitive and/or communicative disability (e.g. due to brain injury).

Subjects were required to be able to follow directions well and demonstrate learning capability.

### Primary and secondary outcomes

The primary outcome measures were the change from baseline in gait spatio-temporal parameters at the end of the training. In particular the spatiotemporal parameters assessed by 3D Gait Analysis was collected at baseline (inclusion) (T0) and after 20 sessions of robot training over an expected average of 5/6 weeks (T1).

The secondary outcome measures were:

1. Participant Satisfaction Questionnaire (10 questions were asked for each subject during and upon the completion of the active participation phase of the treatment) [8];
2. 6 min walking test (6MWT) (the test was administered in indoor and outdoor conditions);
3. Timed Up and Go test (TUG) [8] and Borg Scale [8].

Before, during and after training sessions the subjects performed standardized assessments and complete questionnaires to assess the functional and psychological effects of the exoskeleton (subject’s workload and satisfaction assessed by a VAS). Trained professionals, who were not involved in the research treatment and blind to patients’ treatment, performed all instrumental and clinical assessments.

### 3d-gait analysis

The 3d-gait analysis (GA) was conducted using the following equipment: a 6-camera optoelectronic system with passive markers (SMART 300 DX, BTS, Italy) to measure the kinematics of the movement; 2 TV camera Video systems (BTS, Italy) synchronized with the optoelectronic and force platform systems for video recording. To evaluate the kinematics of each body segment, markers were positioned as described by Helen Hayes [11]. Subjects were asked to walk with EKSO™ robot at their own natural pace (self-selected and comfortable speed), along a 10-meter walkway. At least ten trials were collected for each subject in order to ensure data consistency. All graphs obtained from GA were normalized as a % of the gait cycle. In order to quantify the gait pattern of participants involved in this study, specific software (SmartAnalyzer, BTS, Italy) performed the calculation of some indices (time/distance parameters, joint angles values in specific gait cycle instant) starting from those data.

### Statistical analysis

All the previously defined parameters were computed for each participant. Mean values and standard deviations of all indexes were calculated for each group. Kolomogorov–Smirnov tests were used to verify if the parameters were normally distributed. As this was not the case, we used Wilcoxon’s tests in order to detect significant changes between data at baseline (T0) and endpoint (T1). Statistical significance was set at *p* < 0.05. The Mann–Whitney test was used to compare median scores between groups.

### Training

Three voluntary subjects with chronic spinal cord injury underwent a rehabilitation mobility training consisting of a treatment cycle of 20 sessions of robotic training (50 min for 3/4 times at week) using the EKSO™ system device, according to individually tailored exercise scheduling.

According to Talaty the initial training consisted of learning to sit-to-stand, standing activities within parallel bars, stand-sit transfers, standing balance and stepping skills [12].

Subsequently, training involved learning crutch use placement for balance and limb advancement. The remainder of the training aimed to improve and integrate walking performance with step triggering, coordinating step timing and foot clearance, and safe and effective stopping. Training was specific to each subject and followed their learning pace rather than a predetermined time table. The practice included a robot-assisted walking training at variable speeds for 45/60 min and balance training. All the voluntary recruited into the study had never used any exoskeleton before and they had no familiarity with the device [12].

During training sessions, rest intervals were introduced if required by the participant or suggested by the therapist. The walking is achieved through sensors that detect the weight shifted and activate the individual steps.

Multiple stages of control are used to accomplish the different tasks presented to the controller.

The first stage of control is the Human Machine Interface (HMI). This stage of control is specifically tasked with determining the intended maneuver of the user based on the provided inputs. The second stage is the trajectory generator, which based on the intended maneuver as reported by the HMI along with the current sensor feedback from the device determines what the device should do to accomplish the intended maneuver.

The final stage of control is the low-level controller, which generates the current command for the individual joints to reach the desired motion resulting from the trajectory generation.

This stage is a more classical control method as it includes the closed loop tracking of a desired joint angle by adjusting the commanded current to the motor at the joint.

The complete training was divided into 4 modalities:

1. FirstStep mode a physical therapist actuates steps with a button push. This first mode allow the user or a therapist to move through maneuvers as well as the individual phases of those maneuvers. This simple mode relies entirely on input from the GUI for every transition.
2. ActiveStep mode the user takes control of actuating their steps via buttons on the crutches or walker. This semi-advanced mode, uses the input of the HMI sensors to create these guards; for example, to transition from the right foot step phase to the left foot step phase the advanced HMI looks to see that the right crutch has progressed forward through the arm angle sensor and that the crutch has been loaded.
3. ProStep mode the user achieves the next step by moving their hips forward and shifting them laterally (the device recognizes that the user is in the correct position and steps). In particular *these conditions are met than the HMI identifies that the user has moved their crutch forward and shifted weight onto that forward crutch thus intending to step forward. In addition to these guards, some foot sensor information is evaluated to identify that the feet are correctly loaded to allow for a safe step.*
4. ProStep Plus mode the steps are triggered by the user’s weight shift plus the initiation of forward leg movement. Similar transitions are provided for in the advanced HMI throughout the finite state machine to allow for natural, robust and safe transitioning between states.

The primary benefit provided by the advanced state machine is that instead of requiring direct input by the user, it has the potential to view the entire posture and motion of the user to determine intent. In turn it allows the option to identify the user’s intent by perceiving the small motions that the user makes naturally when trying to accomplish that motion.

Potentially, the advanced HMI can provide a safer user experience by identifying and preventing false state triggers that could be caused even in the simple mode due to an inadvertent button press. By looking at the entire pose of the subject, the HMI can identify postures that do not match with the selected intent of the user and then ask for clarification or completely block them if they are deemed unsafe.

Adjustments of training parameters were done every day by the physical therapist based on the quality of walking (adequate step height during swing phase and adequate knee stability during stance phase), current physical condition (observation of breathing rate and degree of transpiration), and motivation (as verbally indicated by the participant). All changes were made in agreement with the participant [13].

### Ethical aspects

This study was performed in accordance with the Declaration of Helsinki and was approved by the ethics committees of IRCCS San Raffaele Pisana. Informed consent was obtained from all subjects enrolled in this study.

## Results

We screened 10 voluntary subjects and 3 of SCI subjects who satisfied the inclusion criteria. No dropouts were recorded during the training and all subjects fulfilled the protocol (compliant subjects: *N* = 3). The distribution of the study subjects (*N* = 3) by age, gender, and main clinical and demographical characteristics are shown in Table 1. Tables 2 and 3 summarizes the observed mean ± standard deviation for GA (T0 versus T1), as measured on the compliant subjects at T0 (*N* = 3), T1 (*N* =3) (Tables 2 and 3). The results of instrumental assessment showed a statistical improvement in velocity (T0 0,17 ± 0,04 m/s and T1 0,23 ± 0,04 m/s) *p* = 0,0188 and cadence (T0 36,36 ± 7,70 and T1 41,21 ± 4,30). *p* = 0,0120. All results were reported in Tables 2 and 3.

**Table 1:** Clinical characteristics of all subjects

	Age	Gender	Level of lesion	ASIA	Walking
Subject 1	50	Male	D10	A	Prostp + with walker
Subject 2	37	Male	D6	C	Prostp + with walker
Subject 3	21	Female	L1	A	Prostp + with walker

**Table 2:** Observed mean ± standard deviation of 3D gait analysis

	Velocity (m/s)		Cadence (step/min)		Step width (m)		Step length (m) RX		Step length (m) LX	
	T0	T1	T0	T1	T0	T1	TO	T1	T0	T1
Subject 1	0,16 ± 0,01	0,24 ± 0,01	34,2 ± 0,759	40,11 ± 1,58	0,19 ± 0,01	0,19 ± 0,01	0,27 ± 0,01	0,34 ± 0,01	0,24 ± 0,01	0,31 ± 0,01
Subject 2	0,13 ± 0,01	0,19 ± 0,01	33,12 ± 1,98	37,56 ± 1,34	0,19 ± 0,01	0,21 ± 0,01	0,24 ± 0,03	0,27 ± 0,01	0,25 ± 0,01	0,31 ± 0,01
Subject 3	0,22 ± 0,01	0,27 ± 0,02	41,76 ± 0,89	45,96 ± 3,25	0,17 ± 0,01	0,17 ± 0,01	0,29 ± 0,02	0,34 ± 0,02	0,32 ± 0,02	0,34 ± 0,02
Mean	0,17	0,23	36,36	41,21	0,18	0,19	0,27	0,32	0,27	0,32
SD	0,04	0,04	4,70	4,30	0,01	0,02	0,03	0,04	0,04	0,02
*P* values	*p* = 0,0188		*p* = 0,0120		n.s		*p* = 0,0494		n.s

**Table 3:** Observed mean ± standard deviation of 3D gait analysis

	Stance time (% stride) RX		Stance time (% stride) LX		Double support (% stride) RX		Double support (% stride) LX		
	T0	T1	T0	T1	T0	T1	TO	T1	
Subject 1	80,34 ± 1,27	80 ± 0,87	80,09 ± 2,03	78,43 ± 1,54	27 ± 2,93	28,07 ± 2,34	30,78 ± 1,21	28,9 ± 1,64	
Subject 2	81,68 ± 1,31	81,93 ± 2,27	81,36 ± 0,62	83,19 ± 0,7	22,26 ± 11,52	28,27 ± 2,6	40,34 ± 9,82	37,29 ± 6,95	
Subject 3	75,17 ± 1,84	76,8 ± 2,66	76,44 ± 2,24	77,31 ± 1,3	26,56 ± 4,1	28,61 ± 5,07	25,1 ± 1,42	25,64 ± 4,55	
Mean	79,06	79,58	79,30	79,64	25,27	28,32	38,74	30,61	
Sd	3,43	2,25	2,55	3,12	2,61	0,273	18,92	6,01	

Table 4 summarizes the observed mean ± standard deviation for all clinical tests (T0 versus T1), as measured on the compliant subjects at T0 (*N* = 3), T1 (*N* =3) (Tables 2 and 3). In particular the results of Borg scale showed a decrement of −36 % (T0 3 ± 3,464 and T1 1,667 ± 1,155) and the TUG test a decrement of −44 % in time (T0 89 ± 24,25 and T1 56,53 ± 9,036). The analysis of VAS Fatigue (T0 3,667 ± 3,055 and T1 2,667 ± 1,528) and VAS Pain (T0 3,333 ± 4,041 and T1 3,00 ± 3,464) showed a decrement respectively of −27 % and −9 % sign of a good effect of robot training on fatigue and on pain. The analysis of 6MWT indoor showed a improvement of 41 % (T0 45,70 ± 15,54 T192,67 ± 17,16) *p* = 0,0013 and the 6MWT outdoor a improvement of 102,78 % (T0 69,23 ± 28,13 T197,67 ± 20,11) *p* = 0,0378. There were no detrimental changes in vital signs or complaints of lightheadedness with prolonged standing [8]. The subject’s workload and satisfaction assessed by a VAS showed a tendency toward positive feelings regarding the training process. The subjects did clearly feel safe and comfortable with the robot at the end of the training. The analysis of the results of Participant Satisfaction Questionnaire showed that all subjects had strong positive comments regarding the emotional/psychosocial benefits of participating in the trial (Table 5).

**Table 4:** Observed mean ± standard deviation for all clinical tests

	Subject 1	T0	T1	Subject 2	T0	T1	Subject 3	T0	T1	T0 Mean ± SD	T1 Mean ± SD	P
TUG		93	55,6		111	66		63	48	89 ± 24,25	56,53 ± 9,036	n.s.
BORG		7	3		1	1		1	1	3 ± 3,464	1,667 ± 1,155	n.s
VAS FATIGUE		7	4		3	3		1	1	3,667 ± 3,055	2,667 ± 1,528	n.s
VAS PAIN		8	7		1	1		1	1	3,333 ± 4,041	3 ± 3,464	n.s
6MWT Indoor		42,6	90		35	77		69,4	111	45,70 ± 15,54	92,67 ± 17,16	0,0013
6MWT Outdoor		54	92		52	81		101,70	120	69,23 ± 28,13	97,67 ± 20,11	0,0378
10 mWT		79	32		86	30,6		43	28,6	69,33 ± 23,07	30,40 ± 1,709	n.s

**Table 5:** Participants satisfaction questionnaire of the training

Questions:	T0 Mean ± SD	T1 Mean ± SD
Training/learning to use the device is not complicated	4.67 ± 0.58	4.33 ± 0.58
Wearing/adjusting the device is relatively simple	4.33 ± 0.58	4.33 ± 1.15
It was comfortable to exercise with the device	4.67 ± 0.58	4.33 ± 0.58
The usage of the device did not cause considerable pain	5.00 ± 0.00	4.67 ± 0.58
I did not feel excessive fatigue while excessive with the device	4.00 ± 1.00	4.00 ± 1.00
After completing the training period I felt comfortable using the device	4.67 ± 0.58	4.67 ± 0.58
Training with the device diminishes the spasticity in my legs	5.00 ± 0.00	5.00 ± 0.00
I did not have breathing difficulties while training with the device	5.00 ± 0.00	5.00 ± 0.00
I felt improvement in my bowel movement during the training program	3.33 ± 1.53	4.00 ± 1.00
After completing the training I felt safe using the device	3.00 ± 2.00	4.67 ± 0.58

(observed mean ± standard deviation at T0 and T1)

## Discussion

The aim of the present study was to examine for the first time the effects of a training program on the walking ability and quality in SCI individuals, using the EKSO™ robot.

The training protocol was tolerated well by all participants and was performed without difficulties.

All participants improved significantly on functional outcomes, and spatiotemporal measures after 20 sessions of training.

The robot is an automatic device that performs functions normally ascribed to humans or a machine in the form of a human. Robotic devices have been developed to relieve physical therapists from the strenuous and non-ergonomic burden of manual body weight support (BWS). In particular the use of robotic locomotors training devices in the rehabilitation setting could potentially augment recovery of ambulation in people following neurological injury by increasing the total duration of training and reducing the labor-intensive assistance provided by physical therapists. In current clinical practice the gait restoration with a robotic device is an integral part of the rehabilitation program of brain-impaired patients. This innovative robot-rehabilitation program is based on the proven understanding that numerous repetitions of functionally oriented movements can stimulate spinal cord reorganization. Technological innovations provided an opportunity to design interventions that take many key aspects of the stimulation of motor relearning. Moreover for gait training it is then of the most critical importance to walk repetitively in a natural gait similar to over-ground gait and with the correct proprioceptive and exteroceptive feedback [14–17]. Many authors have shown the efficacy of robot-assisted gait training on improving the walking function in several neurological diagnoses but the process aimed at restoring this function in patients with a neurological pathology is challenged by the complexity and variability of these disorders [18]. Scientific evidence showed that four mechanisms may be advocated in order to support the effects of robot assisted gait therapy in gait recovery of neurological disease: providing external proprioceptive cues, enhancing the automatic spinal control of locomotion, improving postural control during walking and promoting reconditioning and muscle strengthening of the lower limbs. Until now, we know that the amount and extent of gait recovery depend on multiple factors, including the level and extent of injury, postinjury medical and surgical care, and rehabilitative interventions. Rehabilitative therapies, such as intense repetitive training (“massed practice”) and locomotor training have been shown to promote recovery after incomplete SCI in humans. Although the mechanisms mediating this recovery are not fully understood, activity-dependent plasticity likely plays a major role. Only few studies were conducted about the change in plasticity or reorganization of CNS and spinal cord of SCI subjects that underwent a robotic rehabilitative training for gait recovery and the results are more confusing. A common focus during rehabilitation after spinal cord injury (SCI) is on promoting improvements in functional walking capacity. In particular locomotor training, both overground and on a treadmill using partial body weight support, has been shown to promote recovery in humans with incomplete SCIs. Evidently, the active exercise paradigm mediates plasticity at multiple levels of the neuraxis including the cortex, descending supraspinal motor pathways, and spinal cord circuitry caudal to injury. In humans, intense repetitive training (massed practice) after a cervical spinal injury and robotic locomotor training after a thoracic spinal injury appear to promote cortical plasticity as cortical map reorganization. As with spontaneously occurring cortical plasticity, the substrates and implications of this activity-dependent cortical reorganization after SCI are unclear. Recent studies have shown that patients can receive positive physical and psychological benefits from robotic training, such as improved walking capacity, improved metabolic performance, and increased activity in the cerebellum. There is level 1 evidence from one Randomized Controlled Trial (RCT) supported by several non-RCTs that intensive locomotor training provided over the sub acute phase in incomplete SCI significantly enhances functional ambulation [19–22]. We found significant changes in 6MWT, and spatiotemporal performances that were relatively the same compared to other studies [23, 24]. We found significant changes in most spatiotemporal measures after robotic gait training. Previous studies showed small increases in cadence, step and stride length, and step-length symmetry but these were not significant [25]. Improvements in walking speed were caused by improvements in step length as well as cadence. The increased walking speed might explain some of the observed changes in other spatiotemporal measures [26]. The analysis of the result of TUG test showed a gain in terms of reduction of time. Changes in TUG time may also depend on impairment and recovery of control in the lower extremity and seem to be functionally related to falls due to reduced force generation and greater postural sway [27].

In addition prolonged immobilization in SCI patients results in systemic de-adaptations, which include: cardiovascular deterioration, urinary dysfunction, gastrointestinal and bowel disorders, respiratory depression and increased frequency of pneumonia and other respiratory tract infections, pressure ulcers, neuropathic pain, musculoskeletal disorders and reduction in loss of bone density, osteoporosis and psychological disorders.

Moreover was already demonstrated that after robotic training the results showed the high variability in recovery pattern among patients. Locomotor training, like any other intervention, it is not expected to affect all patients equally; instead, different treatment responses are expected for different patient subgroups (for exampale the capacity of walking).

Weight bearing and over-ground ambulation has been shown to ameliorate many of these problems. Additionally, there are many psychological and social benefits to standing, including improved selfimage, eye-to-eye interpersonal contact, increased vocational, recreational and daily living independence. As demonstrated, in gait training to walk repetitively in a natural manner similar to the over-ground gait and with the correct proprioceptive and exteroceptive feedback is of the most critical importance [28]. In particular our training protocol where the patient interacts with the robot in modalities ProStep and ProStep Plus can overcome all the limitations about the repetitivity and repeatability of the movement with respect to the human-human interaction. In particular in the Pro Step mode the user achieves the next step by moving their hips forward and shifting them laterally (the device recognizes that the user is in the correct position and steps) and in ProStep Plus mode the steps are triggered by the user’s weight shift plus the initiation of forward leg movement. During our training no adverse events, clinical instability or falls were reported. This prospective study is based on a small study sample at one study site and used no blinding or control group. The study included a selected subgroup of patients, who are not representative of the whole SCI population with regard to age, gender or neurological impairments. Thus, the findings are only relevant for the subgroup at study and cannot be generalized to the whole SCI population.

## Conclusion

The focus on mobility represents one of the most innovative features of this study and makes this research useful in clinical practice. A new studies focused on “open question” about how to control the human-robot interaction can promote recovery in such training conditions must be conducted. The positive effects on improvement in spatiotemporal parameters and clinical assessment of the SCI subjects by the EKSO™ therapy togheter with the lack of side effects strongly suports extending the use of a wearable robot therapy in the recovery and improvement of mobility.

## Abbreviations

6MWT: 6 min walk test
GA: 3D-gait analysis
BWS: body weight support
DGO: driven gait orthosis
RCT: randomized controlled trial
ROM: range of motion
SCI: spinal cord injury
TUG: timed up and go test
